# A218 TRADING ONE PROBLEM FOR ANOTHER? THE EMERGENCE OF LYMPHOCYTIC ESOPHAGITIS IN A PEDIATRIC PATIENT WITH EOSINOPHILIC ESOPHAGITIS AFTER TREATMENT WITH DUPILUMAB

**DOI:** 10.1093/jcag/gwad061.218

**Published:** 2024-02-14

**Authors:** J Strauss, M Brundler, L S McKenzie

**Affiliations:** Pediatrics, University of Calgary Cumming School of Medicine, Calgary, AB, Canada; Pediatrics, University of Calgary Cumming School of Medicine, Calgary, AB, Canada; Pediatrics, University of Calgary Cumming School of Medicine, Calgary, AB, Canada

## Abstract

**Background:**

Eosinophilic esophagitis (EoE) is a Th2 driven chronic, inflammatory disorder defined by esophageal dysfunction and a peak eosinophil count (PEC) ≧ 15 eosinophils/high-power field on biopsy. Dupilumab, a human monoclonal antibody that inhibits IL-4 and IL-13 signaling was recently approved for use in Canada in children with EoE. There is limited data on the histologic abnormalities found with reintroduction of dietary triggers on dupilumab.

**Aims:**

To describe emergence of lymphocytic esophagitis without eosinophilia observed during food reintroduction in a pediatric patient with EoE on dupilumab.

**Methods:**

Case report and literature review.

**Results:**

We present a 14-year-old boy with extensive food allergies, eczema, asthma and treatment refractory EoE. After failing to improve with proton-pump inhibitor (PPI), food elimination diet and topical steroids, he was started on an elemental formula with concomitant PPI. This resulted in symptomatic and histologic remission, however food restriction led to poor quality of life.

The patient was started on dupilumab 200 mg every 2 weeks and had a dramatic improvement in his disease course. Over 2 years on treatment, several allergic foods were reintroduced with normal histologic biopsies. However, two dietary challenges led to the emergence of lymphocytic esophagitis with basal zone hyperplasia (BZH) and dilated intercellular spaces (DIS), but no eosinophilia. Subsequent removal of the dietary antigen and endoscopic reassessment led to normal biopsies, without lymphocytic or eosinophilic infiltration.

Lymphocytic esophagitis is rare in children and the etiology is unknown; it is characterized by increased intraepithelial lymphocytes without eosinophils or neutrophils, and associated spongiosis. The emergence of lymphocytic esophagitis was unexpected and subsequent normalization of histology with removal of an antigen suggests that the lymphocytic pattern observed was driven by the allergic food. This may indicate that IL-4/IL-13 antagonism by dupilumab affects other immune signaling pathways, resulting in a lymphocytic-predominant inflammatory response upon exposure to allergic food antigens.

**Conclusions:**

Dupilumab is a promising new medication in the management of pediatric EoE, particularly in the extremely atopic patient. In our case, the use of dupilumab led to improved antigen tolerance and quality of life. However, the emergence of lymphocytic esophagitis was unexpected, and the natural history and prognosis of this condition is not well defined. This is the first case to describe the emergence of lymphocytic esophagitis in a patient treated for eosinophilic esophagitis with dupilumab, and its subsequent resolution with dietary exclusion.

**Funding Agencies:**

None

